# Prognostic Role of Endocan and Platelet-Derived Growth Factor Isoforms in Metastatic Colorectal Cancer

**DOI:** 10.3390/ijms27062600

**Published:** 2026-03-12

**Authors:** Anıl Yıldız, Melin Aydan Ahmed, Abdulmunir Azizy, Simay Çokgezer, Bedirhan Ulufer, Hilal Oğuz Soydinç, Sanem Karabulut, Didem Taştekin, Burak Şakar, Naziye Ak

**Affiliations:** 1 Department of Medical Oncology, Başakşehir Çam and Sakura City Hospital, İstanbul 34480, Türkiye; anilyildiz@live.com; 2 Department of Medical Oncology, Istanbul University Oncology Institute, İstanbul 34093, Türkiye; melinahmed15@yahoo.com (M.A.A.); munir.azizy@gmail.com (A.A.); simaycokgezer@gmail.com (S.Ç.); bedirhanulufer@hotmail.com (B.U.); drsenemkarabulut@gmail.com (S.K.); didem_doktor@hotmail.com (D.T.); buraksakar@gmail.com (B.Ş.); 3 Department of Basic Oncology, Istanbul University Oncology Institute, İstanbul 34093, Türkiye; hoguz@istanbul.edu.tr

**Keywords:** metastatic colorectal cancer, endocan, platelet-derived growth factor, biomarkers, chemotherapy response, survival

## Abstract

To investigate whether pretreatment serum endocan, platelet-derived growth factor (PDGF)-CC, and -DD levels are elevated in metastatic colorectal cancer (mCRC) patients compared to healthy controls, and to assess their independent associations with chemotherapy response and survival, along with their comparative predictive performance. Adult patients with mCRC receiving systemic chemotherapy combined with an anti-angiogenic agent (bevacizumab or aflibercept) were prospectively enrolled, along with healthy controls. Pretreatment serum samples were collected prior to therapy initiation. Endocan, PDGF-CC, and PDGF-DD levels were measured in duplicate using enzyme-linked immunosorbent assay. Treatment response was evaluated according to Response Evaluation Criteria in Solid Tumors (RECIST) v1.1 and categorized as responders (complete or partial response) and non-responders (stable or progressive disease). Progression-free survival (PFS) and overall survival (OS) were recorded. Median serum levels of endocan (405.5 vs. 269.0 pg/mL), PDGF-DD (499.9 vs. 315.1 pg/mL), and PDGF-CC (2330 vs. 1118 pg/mL) were significantly higher in the mCRC group compared to controls (*p* < 0.001). In multivariable logistic regression, all three biomarkers were independently associated with non-response to chemotherapy [Odds ratio (ORs): 1.10 for endocan, 1.05 for PDGF-DD, 1.05 for PDGF-CC; all *p* < 0.05]. For disease progression, Cox regression showed that higher levels of endocan [Hazard ratio (HR) = 1.04], PDGF-DD (HR = 1.03), and PDGF-CC (HR = 1.04) were significant predictors (all *p* < 0.01). Similar associations were observed for overall mortality (HR = 1.04, 1.02, and 1.02, respectively; all *p* < 0.05). Endocan, PDGF-DD, and PDGF-CC are elevated in mCRC and independently predict poor treatment response and adverse survival outcomes, highlighting their potential as prognostic biomarkers.

## 1. Introduction

Colorectal cancer (CRC) is one of the most common malignancies worldwide and a leading cause of cancer-related mortality [1]. Patients diagnosed with metastatic CRC (mCRC) face a particularly poor prognosis, with five-year survival rates of only around 14–17% [2]. Standard treatment for stage IV disease includes combination cytotoxic chemotherapy (typically oxaliplatin- or irinotecan-based regimens) together with targeted therapy against angiogenesis. Bevacizumab, a monoclonal antibody against vascular endothelial growth factor (VEGF)-A, has demonstrated clear clinical benefit when added to first-line chemotherapy in mCRC, significantly improving progression-free and overall survival (OS) [3]. Likewise, the VEGF-trap aflibercept, which binds VEGF-A, VEGF-B, and placental growth factor, showed in the VELOUR trial that its addition to second-line FOLFIRI chemotherapy modestly but significantly prolonged survival in patients who had progressed after oxaliplatin-based therapy [4]. Despite these advances, not all patients derive meaningful benefit from anti-angiogenic therapy. There are currently no validated predictive biomarkers to identify which mCRC patients are most likely to respond to bevacizumab or other VEGF inhibitors [5]. This lack of predictive markers underscores an unmet need for novel prognostic and predictive biomarkers that could guide personalized treatment selection.

In the search for biomarkers, circulating factors related to tumor angiogenesis and the tumor microenvironment have gained interest. Endocan (endothelial cell-specific molecule-1; ESM1) is a soluble dermatan sulfate proteoglycan secreted by activated vascular endothelial cells. It plays a role in modulating angiogenesis, tumor cell proliferation, and migration [6]. High endocan levels have been correlated with greater tumor size, deeper invasion, lymph node involvement, and the presence of metastases in patients with CRC [7]. A recent umbrella review of meta-analyses reported that cancer patients with higher endocan levels had significantly worse OS, with a combined hazard ratio around 2.5 for mortality compared to those with low endocan [8]. Endocan is known to be induced by pro-angiogenic signals such as VEGF, and it correlates with VEGF activity in angiogenesis-related conditions [9].

Another set of candidate biomarkers are the platelet-derived growth factors (PDGFs). PDGFs are a family of growth factors (PDGF-A, -B, -C, and -D) that form functional homo- or heterodimers (e.g., PDGF-AA, -BB, -CC, -DD) and signal through PDGF receptors (PDGFR-α and PDGFR-β) on cells in the tumor stroma and vasculature [10]. This signaling network is crucial for supporting tumor angiogenesis, stromal cell activation, and metastasis. PDGF-CC can drive tumor growth and angiogenesis through activation of cancer-associated fibroblasts (CAFs), whereas PDGF-DD acting via PDGFR-β stimulates carcinoma cell invasion and metastatic spread [11]. Aberrant PDGF signaling has been implicated in resistance to anti-VEGF therapies—overactivation of PDGFs can facilitate angiogenesis via alternative pathways and promote tumor regrowth despite VEGF inhibition [12]. A recent study noted that serum PDGF-CC levels increase with advancing stage of CRC and metastatic dissemination suggesting PDGF-CC may be a useful marker for predicting disease progression [13]. Similarly, another study reported elevated plasma PDGF-DD levels in CRC patients compared to controls [14], and PDGF-D signaling was further implicated in colorectal carcinogenesis through its effects on gene regulation, proliferation, and PDGFR-β activation [15].

Although anti-angiogenic agents combined with chemotherapy (e.g., bevacizumab or aflibercept) have improved survival outcomes in mCRC, there remains a lack of reliable and clinically accessible biomarkers that can predict treatment response or guide prognosis. Given that endocan may reflect VEGF-driven angiogenic activity, while PDGF-CC and PDGF-DD are involved in alternative stromal activation pathways that may mediate resistance to anti-VEGF therapies, these circulating factors have garnered interest as potential predictive and prognostic biomarkers. However, data on their clinical utility in stage IV colon cancer, particularly in relation to different chemotherapy backbones such as oxaliplatin- or irinotecan-based regimens, are limited. Therefore, this study aimed to evaluate whether baseline serum levels of endocan, PDGF-CC, and PDGF-DD are associated with treatment response and outcomes in patients with mCRC treated with either oxaliplatin- or irinotecan-based chemotherapy.

## 2. Results

### 2.1. Serum Biomarker Levels in Patients with mCRC

A total of 30 age- and sex-matched healthy controls and 82 patients with mCRC were analyzed in the study. Median endocan levels were higher in mCRC group compared to control group (405.5 vs. 269.0 pg/mL, *p* < 0.001). Median PDGF-DD and PDGF-CC levels were elevated in the mCRC group compared to control group (499.9 vs. 315.1 pg/mL, *p* < 0.001; 2330 vs. 1118 pg/mL, *p* < 0.001, respectively) (Table 1). Supplementary Table S1 presents the demographic and clinical characteristics of patients with mCRC. Median serum levels of tumor markers were 26.0 U/mL for carbohydrate antigen (CA) 19-9 and 7.0 ng/mL for carcinoembryonic antigen (CEA). The most common tumor locations were the rectum (43.9%) and rectosigmoid region (14.6%). The majority received oxaliplatin-based chemotherapy (78.0%), while 22.0% received irinotecan-based regimens. Metastases were most frequently observed in the liver (64.6%), lymph nodes (52.4%), and lungs (36.6%). Kirsten rat sarcoma (RAS) viral oncogene homolog (KRAS) mutations were identified in 41.5% of cases, followed by neuroblastoma RAS viral oncogene homolog (NRAS) (15.9%) and B-Raf proto-oncogene serine/threonine kinase (BRAF) (3.7%). Bevacizumab was used in 69.5% of patients. At the 6-month evaluation, 70.7% of patients were classified as responders to chemotherapy. Disease progression occurred in 43.9%, and overall mortality was 80.5%. Median follow-up duration was 29 months (IQR: 17–57).

**Figure 1:**
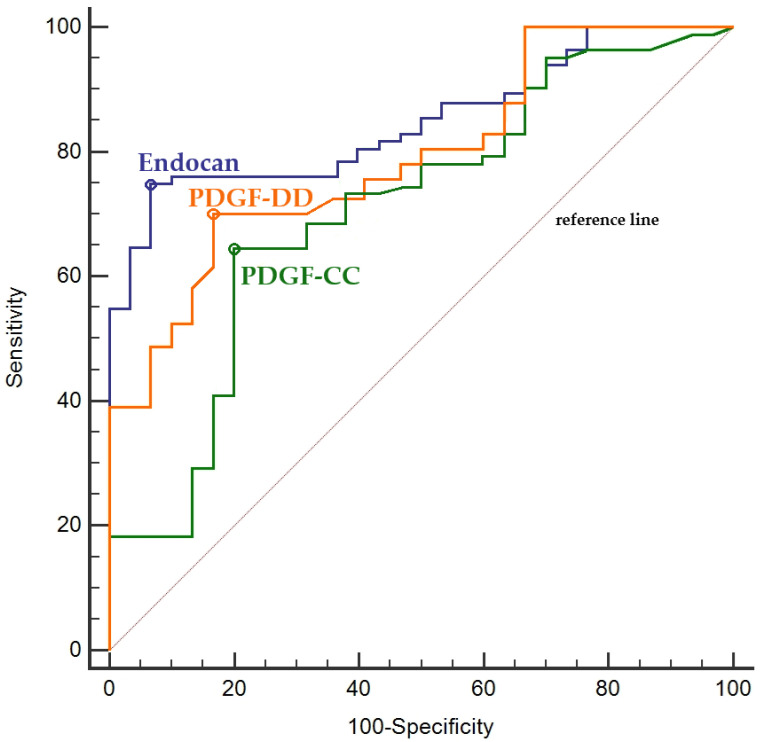
Diagnostic performance of endocan and PDGF isoforms for distinguishing mCRC patients from control subjects. Optimal threshold values are indicated by circular markers on the ROC curves.

Endocan levels demonstrated superior diagnostic performance for predicting mCRC compared with PDGF isoforms [area under the receiver operating characteristic (ROC) curve (AUC): 0.84, 95% CI = 0.74–0.91, Sensitivity: 74.9%, Specificity: 93.0% Threshold value: >324.7 pg/mL for endocan]. In addition, PDGF-DD showed better diagnostic performance than PDGF-CC for predicting mCRC (AUC: 0.74, 95% CI = 0.65–0.82, Sensitivity: 63%, Specificity: 80.2%, Threshold value: >1827 pg/mL for PDGF-CC; AUC: 0.79, 95% CI = 0.62–0.93, Sensitivity: 69.8%, Specificity: 83.2%, Threshold value: >471.7 pg/mL for PDGF-DD) (Figure 1).

### 2.2. Factors Associated with the Best Response to Chemotherapy in Patients with mCR

**Figure 2:**
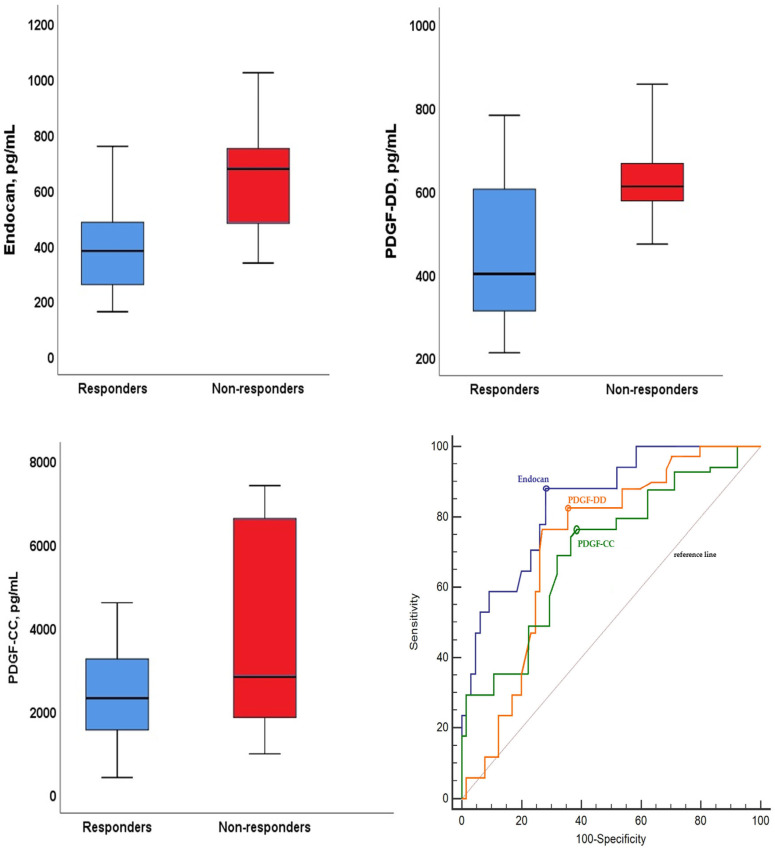
Comparison of serum Endocan, PDGF-DD, and PDGF-CC levels according to best response to chemotherapy, and their diagnostic performance in distinguishing non-responders. Optimal threshold values are indicated by circular markers on the ROC curves.

Among patients with mCRC, demographic, clinical, and molecular characteristics were compared between those who achieved the best response to chemotherapy and those who did not (Supplementary Table S2). A total of 65 patients (79.3%) were classified as responders and 17 patients (20.7%) as non-responders. There were no significant differences between the two groups in terms of age, sex, smoking status, comorbidities, tumor location, chemotherapy regimen, metastasis sites, or mutational status. Median CEA levels were higher in non-responders compared to responders (15.0 vs. 7.0 ng/mL, *p* = 0.047). Clinical outcomes differed markedly between the groups. Non-responders showed significantly higher rates of disease progression (88.2% vs. 32.3%, *p* < 0.001) and mortality (100.0% vs. 75.4%, *p* < 0.001) (Supplementary Table S2). Non-responders exhibited markedly higher median serum concentrations of endocan (675.2 vs. 379.6 pg/mL, *p* < 0.001), PDGF-DD (609.8 vs. 400.0 pg/mL, *p* < 0.001), and PDGF-CC (2576 vs. 2298 pg/mL, *p* < 0.001) compared to responders (Figure 2) (Table 2).

In crude logistic regression analysis, all three biomarkers were significantly associated with non-response to chemotherapy. These associations remained statistically significant in the multivariable logistic regression model, with endocan (OR = 1.10, 95% CI: 1.04–1.15, *p* = 0.001), PDGF-DD [odds ratio (OR) = 1.05, 95% confidence interval (CI): 1.01–1.09, *p* = 0.010], and PDGF-CC (OR = 1.05, 95% CI: 1.01–1.10, *p* = 0.022) identified as independent predictors of chemotherapy non-responders (Table 2). Accordingly, independent of other covariates, a 10 pg/mL increase in endocan levels was associated with a 1.10-fold increase in the odds of non-response to chemotherapy, whereas a 10 pg/mL increase in PDGF-DD and PDGF-CC levels was associated with 1.05-fold increases in the odds of non-response. In the multivariable regression model, all included variables exhibited VIF values < 2.0. Endocan levels demonstrated superior diagnostic performance for distinguishing non-responders compared with PDGF isoforms [AUC: 0.84, 95% CI = 0.74–0.93, Sensitivity: 88.2%, Specificity: 73.2% Threshold value: >456.2 pg/mL for endocan]. PDGF-DD showed better diagnostic performance than PDGF-CC for distinguishing non-responders (AUC: 0.76, 95% CI = 0.66–0.85, Sensitivity: 82.5%, Specificity: 64.8%, Threshold value: >531.3 pg/mL for PDGF-DD; AUC: 0.70, 95% CI = 0.62–0.79, Sensitivity: 76.4%, Specificity: 62.3%, Threshold value: >3590 pg/mL for PDGF-CC) (Figure 2).

### 2.3. Factors Associated with Disease Progression in Patients with mCR

Higher CEA levels, non-response to chemotherapy at 6 months, and poor best response to chemotherapy were significantly associated with disease progression in patients with mCRC. No significant associations were observed for demographic factors, tumor location, chemotherapy type, metastasis sites, or molecular mutations (Supplementary Table S3). Patients with disease progression exhibited significantly higher median serum levels of endocan (482.9 vs. 343.4 pg/mL, *p* < 0.001), PDGF-DD (614.5 vs. 391.0 pg/mL, *p* < 0.001), and PDGF-CC (3089 vs. 1725 pg/mL, *p* < 0.001) compared to those without progression (Table 3).

In the multivariable regression model, all three biomarkers remained independently associated with progression: Endocan [Hazard ratios (HRs): 1.04, *p* < 0.001], PDGF-DD (HR: 1.03, *p* < 0.001), and PDGF-CC (HR: 1.04, *p* = 0.008). Controlling for potential confounders, each 10 pg/mL increment in endocan was independently associated with a 1.04-fold higher hazard of disease progression. Likewise, 10 pg/mL increases in PDGF-DD and PDGF-CC were associated with 1.03- and 1.04-fold higher progression hazards, respectively. In addition, non-response to chemotherapy also emerged as an independent predictor (HR: 2.18, *p* = 0.008), while the effect of CEA levels was no longer statistically significant after adjustment. In the multivariable regression model, all included variables exhibited VIF values < 2.0. (Table 3).

**Figure 3:**
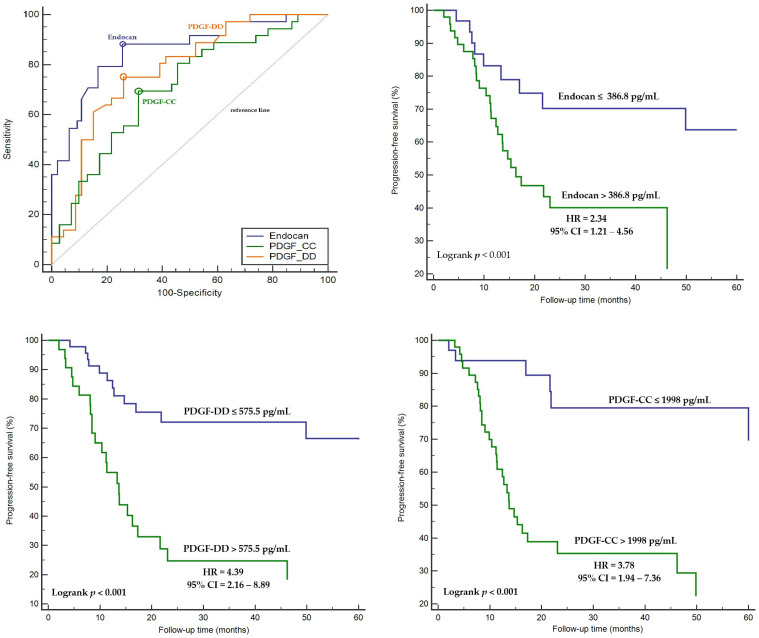
Diagnostic performance of endocan, PDGF-DD, and PDGF-CC in predicting disease progression, Kaplan–Meier survival analysis stratified by biomarker-specific threshold values associated with disease progression risk. Optimal threshold values are indicated by circular markers on the ROC curves.

Endocan levels demonstrated superior diagnostic performance for predicting disease progression compared with PDGF isoforms (AUC: 0.85, 95% CI = 0.76–0.94, Sensitivity: 88.7%, Specificity: 74.6% Threshold value: >386.8 pg/mL for endocan). PDGF-DD showed better diagnostic performance than PDGF-CC for predicting disease progression (AUC: 0.78, 95% CI = 0.68–0.87, Sensitivity: 75.0%, Specificity: 74.6%, Threshold value: >575.5 pg/mL for PDGF-DD; AUC: 0.72, 95% CI = 0.64–0.80, Sensitivity: 69.4%, Specificity: 69.8%, Threshold value: >1998 pg/mL for PDGF-CC) (Figure 3). Patients with serum endocan and PDGF isoform levels above the identified threshold values had a higher risk of disease progression (Figure 3).

### 2.4. Factors Associated with Mortality in Patients with mCR

Elevated CEA levels, rectosigmoid tumor location, non-response to chemotherapy, poor best response to chemotherapy, and disease progression were significantly associated with mortality. No significant associations were found for age, gender, chemotherapy type, metastatic sites, or mutation status (Supplementary Table S4). Median biomarker concentrations were significantly higher in deceased patients compared to survivors: Endocan (453.4 vs. 240.4 pg/mL, *p* < 0.001), PDGF-DD (536.2 vs. 280.8 pg/mL, *p* = 0.001), and PDGF-CC (2872 vs. 1439 pg/mL, *p* = 0.005) (Table 4). All three biomarkers remained independently associated with increased mortality: Endocan (HR: 1.04, 95% CI: 1.02–1.06, *p* < 0.001), PDGF-DD (HR: 1.02, 95% CI: 1.01–1.04, *p* = 0.003), and PDGF-CC (HR: 1.02, 95% CI: 1.01–1.05, *p* = 0.018). Controlling for other covariates, each 10 pg/mL increment in endocan was independently associated with a 1.03-fold increased hazard of death, whereas comparable increases in PDGF-DD and PDGF-CC were associated with 1.02-fold higher mortality hazards. Disease progression was also a strong and independent predictor of mortality (HR: 2.79, 95% CI: 1.33–5.88, *p* = 0.007). CEA levels and tumor location did not retain significance after adjustment. In the multivariable regression model, all included variables exhibited VIF values < 2.0 (Table 4).

**Figure 4:**
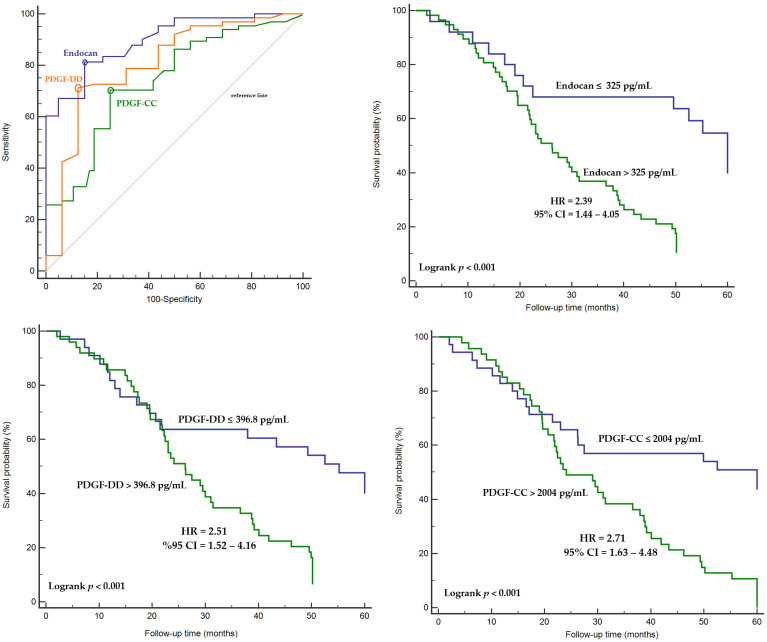
Diagnostic performance of endocan, PDGF-DD, and PDGF-CC in predicting mortality, Kaplan–Meier survival analysis stratified by biomarker-specific threshold values associated with mortality risk. Optimal threshold values are indicated by circular markers on the ROC curves.

Endocan levels demonstrated superior diagnostic performance for predicting disease progression compared with PDGF isoforms (AUC: 0.88, 95% CI = 0.78–0.98, Sensitivity: 81.7%, Specificity: 85.0% Threshold value: >325 pg/mL for endocan). PDGF-DD showed better diagnostic performance than PDGF-CC for predicting disease progression (AUC: 0.83, 95% CI = 0.73–0.90, Sensitivity: 71.2%, Specificity: 87.5%, Threshold value: >396.8 pg/mL for PDGF-DD; AUC: 0.74, 95% CI = 0.62–0.86, Sensitivity: 70.0%, Specificity: 75%, Threshold value: >2004 pg/mL for PDGF-CC) (Figure 4). Patients with serum endocan and PDGF isoform levels above the identified cut-off values had a higher risk of disease progression (Figure 4).

### 2.5. Associations Between Serum Endocan, PDGF Isoforms, and Tumor Markers

Correlation analysis revealed significant positive associations between endocan and PDGF-DD (r = 0.417, *p* < 0.001), as well as endocan and PDGF-CC (r = 0.407, *p* < 0.001). PDGF-DD also showed a positive correlation with PDGF-CC (r = 0.551, *p* < 0.001). In addition, endocan demonstrated significant positive correlations with CA 19-9 (r = 0.319, *p* = 0.048) and CEA (r = 0.332, *p* = 0.006). No significant correlations were observed between PDGF isoforms and CA 19-9 or CEA (Table 5).

## 3. Discussion

Although endothelial and angiogenesis-related pathways have been extensively studied in CRC, the clinical relevance of circulating biomarkers such as endocan (ESM1), PDGF-CC, and PDGF-DD remains incompletely characterized. Prior studies have typically evaluated these ligands in isolation, with limited attempts to compare their predictive and prognostic performance or to investigate potential mechanistic overlap. Furthermore, head-to-head analyses of the endocan and PDGF axes in the same patient cohort are virtually absent from the literature. Addressing this gap, our study provides a comparative assessment of serum endocan, PDGF-CC, and PDGF-DD levels in mCRC patients, evaluating their associations with chemotherapy response, PFS, and OS. All three biomarkers were significantly elevated in patients compared to healthy controls and demonstrated independent associations with both treatment response and survival outcomes. Among them, endocan emerged as the strongest overall predictor across all clinical endpoints, followed by PDGF-DD. In addition, endocan displayed positive correlations with both PDGF isoforms. These findings offer new insight into the distinct yet partially overlapping biological roles of these angiogenesis-related markers and their potential clinical utility in mCRC.

In the present study, patients with mCRC exhibited significantly higher serum endocan levels compared with healthy controls. Moreover, elevated endocan levels were associated with an increased risk of disease progression and mortality, highlighting its potential value as a prognostic biomarker in advanced CRC. Ji et al. reported that endocan expression was elevated in both tumor tissue and serum samples of CRC patients, and that higher levels were associated with more advanced disease stages and increased mortality risk [16]. Similarly, Jiang et al. found that serum ESM1 levels were significantly higher in CRC patients than in healthy individuals and were positively correlated with poor histological differentiation, deeper tumor invasion, lymph node metastasis, and TNM stage. Their multivariate analysis further demonstrated that high serum ESM1 was independently associated with shorter OS, with a fourfold increase in mortality risk [7]. Mechanistic studies also support the involvement of endocan in tumor progression. Yang et al. showed that overexpression of ESM1 in CRC cell lines enhanced tumor cell migration, invasion, angiogenesis, and colony formation. These effects were mediated through activation of the phosphoinositide 3-kinase (PI3K)/protein kinase B (Akt)/mechanistic target of rapamycin (mTOR) signaling pathway, which modulated downstream angiogenic and proliferative factors such as VEGF, hypoxia-inducible factor 1 alpha (HIF-1α), cyclooxygenase-2 (COX-2), and matrix metalloproteinases (MMPs) [17]. Furthermore, a recent single-cell ribonucleic acid (RNA) sequencing study by Xie et al. identified ESM1 as a tip-cell-specific marker within the tumor endothelium of CRC. Their findings revealed a VEGFA–kinase insert domain receptor (KDR)–ESM1 positive feedback loop promoting endothelial activation, angiogenesis, and tumor progression. The density of tip cells was correlated with worse prognosis, and disruption of this loop via programmed cell death protein 1 blockade significantly suppressed angiogenic signaling. These results offer a compelling biological framework for interpreting our clinical observation that elevated circulating endocan levels reflect aggressive tumor biology and angiogenesis in CRC [18].

To our knowledge, this is one of the few clinical studies to report an independent association between baseline serum endocan/ESM1 levels and chemotherapy response in patients with mCRC. Mechanistically, endocan is a downstream product of endothelial activation regulated by VEGF and hypoxia and is therefore closely linked to the biology of angiogenesis-targeted therapies. Preclinical studies have shown that endocan can directly activate PDGFRA and enhance downstream MYC proto-oncogene protein signaling, contributing to radioresistance. Furthermore, high ESM1 expression has been associated with reduced sensitivity to bevacizumab, while the addition of an anti-ESM1 monoclonal antibody has been reported to improve tumor response, suggesting a potential role for the endocan axis in modulating treatment efficacy, particularly in regimens combining anti-VEGF agents with oxaliplatin or irinotecan [19]. Nevertheless, contradictory evidence exists. In a translational sub-study of the ITACa phase III trial, baseline and early-treatment (week 8) serum levels of angiogenesis-related proteins, including endocan, were assessed using multiplex and single enzyme-linked immunosorbent assay (ELISA) platforms (Bio-Plex/Luminex) in 58 mCRC patients receiving first-line bevacizumab plus FOLFOX4 or FOLFIRI. Treatment response was evaluated according to Response Evaluation Criteria in Solid Tumors (RECIST) version 1.1, and statistical models accounted for chemotherapy regimen as a covariate. In that study, while VEGF-C, macrophage-derived chemokine, and epidermal growth factor were significantly associated with objective response rate, baseline endocan levels were not. Moreover, early changes in endocan levels were not among the strongest modulators of clinical response, which were more prominently linked to angiopoietin-2 and interleukin-8 modulation [20]. These conflicting findings highlight the complexity of the angiogenic microenvironment and suggest that while endocan may be biologically relevant, its clinical predictive value for treatment response remains to be further validated in larger, prospective cohorts.

Serum levels of both PDGF-CC and PDGF-DD were significantly higher in patients with mCRC compared to healthy controls, and elevated levels of these biomarkers were associated with poor treatment response and worse prognosis. Although studies directly evaluating circulating PDGF-CC and PDGF-DD levels in CRC are limited, existing transcriptomic and tissue-level data support their upregulation. Manzat-Saplacan et al. demonstrated significantly higher PDGFC messenger RNA expression in the peripheral blood of CRC patients compared with individuals without colonic pathology [21]. Similarly, Yamauchi et al. reported increased PDGFC mRNA expression in tumor tissue compared to adjacent normal mucosa, along with distinct protein-level differences on immunohistochemical analysis [22]. A similar pattern has been reported for PDGF-DD in CRC patients. Jiang et al. demonstrated significantly higher PDGF-D expression in CRC tissues relative to matched normal mucosa, confirmed by both immunohistochemical staining and quantitative polymerase chain reaction (PCR) analysis [23]. Supporting this, a conference report indicated that preoperative plasma levels of PDGF-DD were elevated in patients with colorectal cancer compared to those with benign colonic diseases [14]. Similar findings have been reported in various other malignancies [24,25,26]. These elevations may reflect the underlying biological roles of these ligands in tumor progression and microenvironmental remodeling.

A prospective cohort presented at ESMO Asia 2022 identified serum PDGF-CC levels ≥ 365 pg/mL as an independent predictor of poor chemotherapy response in CRC patients [13]. Additionally, transcriptomic and immunohistochemical analyses have consistently associated PDGFC overexpression with unfavorable overall and relapse-free survival outcomes, with hazard ratios exceeding 3.0 in multivariable models [27,28]. To date, however, no study has concurrently evaluated PDGF-DD in relation to both treatment response and prognosis in CRC or directly compared its diagnostic performance with PDGF-CC. In the present study, both biomarkers remained independently associated with chemotherapy non-response and adverse clinical outcomes. Notably, PDGF-DD exhibited superior discriminatory performance for predicting treatment non-response, whereas both biomarkers demonstrated comparable prognostic capacity for progression-free survival (PFS) and overall mortality. These findings may reflect fundamental biological differences between the two ligands. PDGF-DD appears to more directly capture biological programs that influence immediate treatment response, such as epithelial–mesenchymal transition (EMT), invasiveness, and survival signaling downstream of PDGFR-β. In contrast, PDGF-CC may better reflect a broader stromal activation state, including CAF-mediated extracellular matrix remodeling, immunosuppression, and angiogenesis via PDGFR-αα and -αβ signaling [29]. Mechanistically, PDGF-DD has been shown to induce EMT and tumor invasiveness through activation of the Notch receptor 1/TWIST family bHLH transcription factor 1 axis, alongside increased expression of VEGF, MMPs, and Cyclin D1 [30]. Given the preferential expression of PDGFR-β in pericytes and subsets of activated fibroblasts, elevated PDGF-DD may indicate a pericyte-enriched, vascularized stromal phenotype—commonly associated with reduced drug penetration and rapid disease adaptation [10]. Moreover, PDGF-DD may engage the signal transducer and activator of transcription 3 (STAT3) signaling cascade, a key regulatory node in therapeutic resistance. In preclinical models of pancreatic ductal adenocarcinoma, PDGFD upregulation—mediated via reversible promoter demethylation—led to autocrine and paracrine STAT3 activation, which in turn promoted resistance to gemcitabine by inducing deoxyribonucleic acid (DNA) repair machinery such as ribonucleotide reductase regulatory subunit M1 [31]. Similarly, in advanced urothelial carcinoma, a PDGF-DD–STAT3 feedback loop was associated with poor clinical outcomes following immune checkpoint blockade, and PDGF-DD was proposed as a biomarker for identifying patients likely to benefit from STAT3-targeted combination therapies [32]. Although these findings derive from tumor types other than CRC, they offer converging mechanistic evidence that PDGF-DD/PDGFD can interface with STAT3-driven resistance networks—supporting the hypothesis that elevated pretreatment PDGF-DD levels may identify mCRC patients at risk of early treatment failure and unfavorable prognosis.

**Figure 5:**
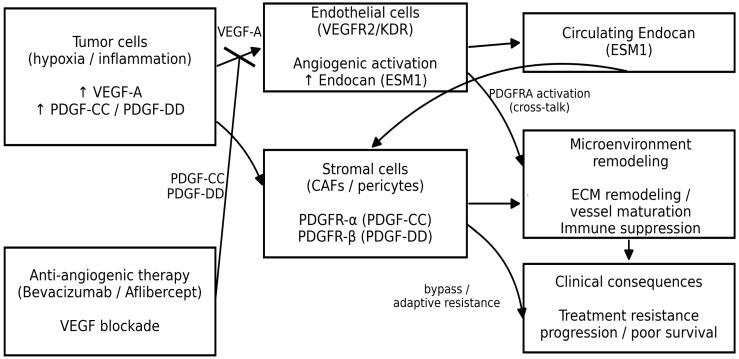
Conceptual schematic summarizing the proposed biological interplay between VEGF signaling, Endocan (ESM1), PDGF-CC/PDGF-DD signaling, and stromal activation, which may contribute to angiogenesis, microenvironment remodeling, treatment resistance, and outcomes in mCRC. Abbreviations: CAFs, cancer-associated fibroblasts; ECM, extracellular matrix; mCRC, metastatic colorectal cancer.

Another important finding of this study was that serum endocan outperformed PDGF ligands in diagnostic accuracy for all endpoints. This difference may reflect stronger integration of endocan into tumor–stroma crosstalk and vascular/stromal activation within the tumor microenvironment. In line with this hypothesis, endocan levels showed a positive correlation with PDGF isoforms. Endocan is increasingly viewed as a marker of activated, sprouting tumor endothelium; single-cell work in CRC identifies ESM1-enriched tip endothelial cells and a VEGFA–KDR–ESM1 positive feedback loop, consistent with coordinated upregulation in highly angiogenic tumors [18]. In parallel, PDGF isoforms reflect tumor–stroma crosstalk (CAF/perivascular activation, matrix remodeling, vessel maturation) and are frequently co-driven by hypoxia/inflammation in aggressive disease; PDGFC in particular is linked to CAF-rich, poor-prognosis biology in colon cancer [27]. A mechanistic bridge is also biologically plausible: endothelial-secreted endocan has been shown to activate PDGFRA signaling in cancer models, providing a concrete template for coordinated elevations of endocan with PDGF-axis activity [19]. PDGF signaling can participate in growth-factor network redundancy/bypass, which may co-amplify VEGF-endothelial programs indexed by endocan [33], because PDGF ligands may also be influenced by platelet activation in serum, a component of the correlation could reflect a coupled “vascular/platelet activation” state in advanced cancer [34]. Figure 5 depicts a schematic representation of the potential interactions between VEGF signaling pathways, endocan (ESM1), PDGF isoforms, and stromal activation, highlighting their possible effects on clinical endpoints.

Recent evidence also supports the prognostic relevance of simple circulating blood-based indices reflecting systemic inflammation, platelet activation, and nutritional status in CRC. For instance, a large retrospective study demonstrated that the combined preoperative platelet-to-albumin ratio (PAR) and cancer inflammation prognostic index (CIPI) independently predicted postoperative overall survival and improved nomogram-based risk stratification [35]. Similarly, a novel immune-nutritional prognostic ratio integrating immune and nutritional parameters (INPR) was reported to predict long-term survival in stage I–III CRC [36]. Although these indices were developed primarily in non-metastatic/postoperative settings, they underscore the broader value of easily accessible circulating markers capturing host-tumor interactions. Mechanistically, tumor microenvironment remodeling and treatment resistance are increasingly linked to metabolic reprogramming and its crosstalk with immune suppression [37], providing a conceptual framework for how angiogenesis- and stroma-related circulating markers (including endocan and PDGF isoforms) may reflect an adaptive, therapy-resistant microenvironment.

Although the adjusted odds ratios and hazard ratios per 10 pg/mL increase may appear numerically modest, they should be interpreted in the context of the measurement scale of these continuous biomarkers (pg/mL) and the wide concentration ranges observed in mCRC. In clinical practice, risk accumulates across clinically meaningful differences in biomarker levels rather than per 10 pg/mL increment alone. Furthermore, the ROC-derived thresholds and Kaplan–Meier stratification in our cohort demonstrated clear separation between higher- and lower-risk groups for non-response, progression, and mortality. Therefore, the most clinically meaningful application is risk stratification (e.g., above vs. below an exploratory threshold or by biomarker quantiles) and/or integration into multivariable risk scores together with readily available clinicopathologic factors (e.g., metastatic burden, RAS/BRAF status, treatment backbone) and tumor markers such as CEA. Such approaches could support baseline counseling and guide the intensity of radiologic surveillance or early treatment reassessment.

Several limitations should be acknowledged. First, this was a single-center study with a moderate sample size, which may limit generalizability and restrict the precision of subgroup analyses. Second, biomarker levels were only assessed at baseline; dynamic changes in response to treatment—which may have prognostic or predictive value—were not captured. The study also did not assess potential interactions between endocan and PDGF signaling axes, which may converge on shared downstream pathways such as PI3K/Akt/mTOR or STAT3. Third, treatment exposure was not randomized; the choice between oxaliplatin- and irinotecan-based chemotherapy backbones followed routine clinical practice, and although most patients received bevacizumab, anti-angiogenic therapy was not uniformly administered, raising the possibility of confounding by indication when comparing treatment-stratified performance. Fourth, due to the limited sample size, internal validation of the ROC-derived cutoff values for diagnostic and outcome prediction analyses using resampling techniques (e.g., bootstrapping or cross-validation) was not performed. Therefore, these thresholds and their associated performance estimates should be interpreted as preliminary and exploratory. Finally, external validation in an independent cohort, preferably with predefined cutoffs and harmonized assay conditions, is required before clinical implementation.

## 4. Materials and Methods

This prospective observational study enrolled adult patients with mCRC who were evaluated at the Gastrointestinal Tumors Outpatient Clinic, Oncology Institute, Istanbul University, between August 2020 and December 2025. The study was conducted in accordance with the Declaration of Helsinki and was approved by the Istanbul University Ethics Committee (Date: 17 July 2020, Decision No: 117098). Written informed consent was obtained from all participants prior to inclusion in the study.

### 4.1. Study Population

Eligible participants were adults (≥18 years) with a histologically confirmed diagnosis of colorectal adenocarcinoma and radiologically verified stage IV disease. Tumors were staged according to the American Joint Committee on Cancer (AJCC) TNM classification (7th edition) [38]. Inclusion criteria further comprised patients aged 18 years or older who were scheduled to receive first-line systemic chemotherapy in combination with an anti-angiogenic agent (bevacizumab or aflibercept), and who provided written informed consent and agreed to donate baseline serum samples for biomarker analysis. All patients received either an oxaliplatin-based regimen (e.g., FOLFOX) or an irinotecan-based regimen (e.g., FOLFIRI) as part of their treatment protocol.

Exclusion criteria were: age < 18 years; prior systemic therapy for metastatic disease; concurrent active malignancy other than colorectal cancer; severe or uncontrolled comorbidities that could confound biomarker levels or preclude standard systemic therapy (e.g., decompensated heart failure, advanced hepatic failure, end-stage renal disease, or recent major cerebrovascular event); active autoimmune or chronic inflammatory disease requiring systemic immunosuppressive treatment; clinically significant active infection at baseline (defined as infection requiring systemic antimicrobial therapy and/or hospitalization within the preceding 2 weeks); and unavailability of baseline serum samples or unsuccessful biomarker measurements. Patients receiving predefined medication classes considered to substantially affect angiogenesis-related biomarkers—such as chronic systemic corticosteroids, biologic agents, or other long-term immunosuppressants—were excluded. Common chronic conditions such as hypertension and type 2 diabetes mellitus were not considered exclusion criteria, provided that they were medically controlled and clinically stable.

A control group of healthy volunteers was also included for comparative biomarker analysis. Controls were adults aged ≥18 years with no history of malignancy, chronic illness, or regular medication use, who voluntarily agreed to participate and provided baseline serum samples.

### 4.2. Data Collection

Baseline demographic and clinical variables were recorded at enrollment, including age, sex, smoking status, comorbidities, primary tumor location, chemotherapy backbone (oxaliplatin- vs. irinotecan-based), anti-angiogenic agent used, metastatic sites, and lymph node involvement. Pretreatment serum biomarkers—CEA, CA 19-9, endocan, PDGF-CC, and PDGF-DD—were measured prior to the first treatment dose. Molecular characteristics (KRAS, NRAS, and BRAF mutation status) were retrieved from routine clinical testing performed on formalin-fixed paraffin-embedded tumor tissue using polymerase chain reaction (PCR)-based methods on formalin-fixed paraffin-embedded (FFPE) tumor tissue samples. Treatment-related variables included adverse events/toxicity and radiologic response at 6 months as well as best response to chemotherapy. Disease progression and death were recorded during follow-up. Follow-up time was calculated from treatment initiation to last contact or death.

### 4.3. Laboratory Measurements

Venous blood samples were obtained after an overnight fast (≥8 h) at baseline, prior to the initiation of systemic therapy. Routine blood parameters were analyzed using automated laboratory analyzers (Mindray MC6800; Mindray, Shenzhen, China). For the measurement of serum endocan, PDGF-CC, and PDGF-DD, blood samples were allowed to stand at room temperature for 30 min and were then centrifuged at 4000 rpm for 10 min. The separated serum samples were stored at −80 °C until the completion of sample collection.

Human PDGF-DD and PDGF-CC levels were measured using commercially available Quantikine^TM^ enzyme-linked immunosorbent assay (ELISA) kits (Catalog Numbers DDD00 and DCC00, R&D Systems, Minneapolis, MN, USA) following the manufacturers’ protocols. Human Endocan (ESM1) concentrations in serum samples were determined using a commercially available sandwich ELISA kit (Catalog Number ab213776, Abcam, Cambridge, UK) according to the manufacturer’s instructions.

Levels of CEA and CA19-9 were measured in the institutional central laboratory using automated immunoassay systems. All laboratory personnel were blinded to patient clinical outcomes to prevent measurement bias.

### 4.4. Outcomes

Tumor response was assessed according to RECIST version 1.1. Computed tomography (CT) imaging was performed at baseline and every 12 weeks. Best response to chemotherapy were recorded and categorized as complete response (CR), partial response (PR), stable disease (SD), or progressive disease (PD).

PFS was defined as the time from treatment initiation to radiologically confirmed progression or death from any cause, whichever occurred first. OS was defined as the time from treatment initiation to death from any cause. Patients without an event at data cutoff were censored at the date of last follow-up.

### 4.5. Statistical Analysis

All analyses were conducted using IBM SPSS Statistics for Windows, version 25.0 (IBM Corp., Armonk, NY, USA). The normality of distribution for continuous variables was assessed using the Kolmogorov–Smirnov test. Normally distributed variables were presented as mean ± standard deviation (SD), and non-normally distributed variables as median and interquartile range (IQR: 25th–75th percentiles). Comparisons between two independent groups were performed using Student’s *t*-test for normally distributed variables, and the Mann–Whitney U test for non-normally distributed variables. For comparisons involving more than two groups, one-way analysis of variance (ANOVA) or the Kruskal–Wallis test was used, depending on the distribution. Categorical variables were expressed as counts and percentages, and compared using the Chi-square or Fisher’s exact test, as appropriate. Factors associated with chemotherapy non-response (best response to chemotherapy: SD/PD vs. CR/PR) were assessed using univariate and multivariate logistic regression analyses, with results presented as ORs and 95% CIs. Prognostic factors for PFS and OS were evaluated using univariable and multivariable Cox regression analyses, with results presented as HRs and 95% CIs. Variables with clinical relevance or a univariable *p*-value < 0.05 were entered into multivariable logistic and Cox regression models. The proportional hazards assumption was evaluated using log-minus-log survival plots and Schoenfeld residuals. Multivariable models were adjusted for covariates such as age, sex, smoking status, presence of comorbidity, chemotherapy backbone (oxaliplatin- vs. irinotecan-based), metastasis area, mutation status, and/or anti-angiogenic agent type, as detailed in the respective table footnotes. Multicollinearity among variables was assessed using the variance inflation factor (VIF), and a threshold of VIF > 5.0 was used to define multicollinearity [39]. ROC curve analyses were performed to evaluate the discriminatory performance of endocan, PDGF-CC, and PDGF-DD for (i) distinguishing mCRC patients from healthy controls, (ii) predicting chemotherapy non-response, (iii) predicting disease progression, and (iv) predicting mortality. The AUC with 95% CIs was reported. Optimal cut-off values were determined by maximizing the Youden index (sensitivity + specificity − 1). Due to the sample size, these ROC-derived thresholds were not internally validated (e.g., bootstrapping or cross-validation) and should therefore be considered exploratory. Time-to-event outcomes, including PFS and OS, were estimated using the Kaplan–Meier method and compared between groups using the log-rank test. A two-tailed *p*-value < 0.05 was considered statistically significant for all analyses.

## 5. Conclusions

Metastatic CRC patients exhibited elevated endocan, PDGF-DD, and PDGF-CC levels, and increased release of these biomarkers was independently associated with both poorer chemotherapy response and adverse survival outcomes. Across all endpoints, endocan showed the strongest predictive performance, followed by PDGF-DD. The positive correlations between endocan and PDGF isoforms further suggest partially overlapping endothelial–stromal activation biology in advanced disease. These findings support the potential clinical utility of baseline endocan and PDGF isoforms—particularly endocan and PDGF-DD—for early risk stratification in mCRC.

## Figures and Tables

**Table 1:** Comparison of demographic characteristics and serum biomarker levels between control subjects and patients with mCRC.

Variables	Control	mCRC	*p*
	n = 30	n = 82	
Age, years	58.4 ± 12.7	59.2 ± 11.6	0.747
<65, n (%)	22 (73.3)	56 (68.3)	0.607
≥65, n (%)	8 (26.7)	26 (31.7)	
Gender, n (%)			
Male	20 (66.7)	51 (62.2)	0.664
Female	10 (33.3)	31 (37.8)	
Smoking, n (%)	12 (40.0)	36 (43.9)	0.712
Endocan, pg/mL	269.0 (171.1–311.1)	405.5 (273.6–531.1)	<0.001 *
PDGF-DD, pg/mL	315.1 (159.9–396.1)	499.9 (342.1–632.3)	<0.001 *
PDGF-CC, pg/mL	1118 (678–1697)	2330 (1532–3258)	<0.001 *

Data are mean ± SD or median (IQR), or number (%). * *p* < 0.05 indicates statistical significance. Abbreviations: mCRC, metastatic colorectal cancer; SD, standard deviation; IQR, interquartile range; PDGF-DD, platelet-derived growth factor-DD; PDGF-CC, platelet-derived growth factor-CC.

**Table 2:** Factors associated with non-response to chemotherapy in mCRC patients.

Variables	Best Response to CTx		Crude Regression		Multivariable Model		VIF
	Responders n = 65	Non-Responders n = 17	OR (95% CI)	*p*	OR (95% CI)	*p*	
CEA, ng/mL	7.0 (2.0–21.0)	15.0 (4.0–34.0)	1.04 (1.01–1.10)	0.045 *	1.04 (0.99–1.03)	0.834	1.03
Endocan, pg/mL	379.6 (257.8–482.9)	675.2 (468.5–780.4)	1.08 (1.04–1.12)	<0.001 *	1.10 (1.04–1.15)	0.001 *	1.11
PDGF-DD, pg/mL	400.0 (310.9–603.6)	609.8 (575.5–665.2)	1.05 (1.02–1.08)	0.004 *	1.05 (1.01–1.09)	0.010 *	1.40
PDGF-CC, pg/mL	2298 (1532–3239)	2876 (1860–6610)	1.04 (1.01–1.07)	0.014 *	1.05 (1.01–1.10)	0.022 *	1.37
					Nagelkerke R^2^ = 0.49		

Data are mean ± standard deviation or median (IQR). Age, gender, smoking, presence of comorbidity, tumor location, chemotherapy type, metastasis area, mutation status, and anti-angiogenic agent type were adjusted in multivariable analyses. * *p* < 0.05 indicates statistical significance. For endocan, PDGF-DD, and PDGF-CC, OR (95% CI) are expressed per 10 pg/mL increase. Abbreviations: CEA, carcinoembryonic antigen; CI, confidence interval; CTx, chemotherapy; OR, odds ratio; PDGF-DD, platelet-derived growth factor-DD; PDGF-CC, platelet-derived growth factor-CC.

**Table 3:** Factors associated with disease progression in mCRC patients.

Variables	Disease Progression		Crude Regression		Multivariable Model		VIF
	No n = 46	Yes n = 36	HR (95% CI)	*p*	HR (95% CI)	*p*	
CEA, ng/mL	7.0 (2.2–23.2)	18.0 (5.0–35.5)	1.03 (1.02–1.07)	0.047 *	1.02 (0.98–1.08)	0.260	1.03
Best response to CTx							1.52
Responders	44 (95.7)	21 (58.3)	ref		ref		
Non-responders	2 (4.3)	15 (41.7)	3.98 (2.03–7.81)	<0.001 *	2.18 (1.06–4.75)	0.008 *	
Endocan, pg/mL	343.4 (222.7–467.6)	482.9 (366.4–692.1)	1.04 (1.02–1.06)	<0.001 *	1.04 (1.02–1.06)	<0.001 *	1.43
PDGF-DD, pg/mL	391.0 (291.7–533.3)	614.5 (487.3–669.4)	1.03 (1.01–1.04)	<0.001 *	1.03 (1.01–1.04)	<0.001 *	1.44
PDGF-CC, pg/mL	1725 (1217–2743)	3089 (2280–4456)	1.03 (1.01–1.05)	<0.001 *	1.04 (1.01–1.06)	0.008 *	1.36
					−2 Log Likelihood = 245.8		

Data are mean ± standard deviation or median (IQR), or number (%). Age, gender, smoking, presence of comorbidity, tumor location, chemotherapy type, metastasis area, mutation status, and anti-angiogenic agent type were adjusted in multivariable analyses. * *p* < 0.05 indicates statistical significance. For endocan, PDGF-DD, and PDGF-CC, HR (95% CI) are expressed per 10 pg/mL increase. Abbreviations: CEA, carcinoembryonic antigen; CI, confidence interval; CTx, chemotherapy; HR, hazard ratio; PDGF-DD, platelet-derived growth factor-DD; PDGF-CC, platelet-derived growth factor-CC.

**Table 4:** Factors associated with mortality in mCRC patients.

Variables	Survival		Crude Regression		Multivariable Model		VIF
	Alive n = 16	Deceased n = 66	HR (95% CI)	*p*	HR (95% CI)	*p*	
CEA, ng/mL	3.5 (2.0–7.2)	9.5 (4.0–33.2)	1.03 (1.01–1.06)	0.007 *	1.01 (0.98–1.03)	0.118	1.04
Tumor location, n (%)							1.05
Rectum	10 (62.5)	26 (39.4)	ref		ref		
Rectosigmoid	1 (6.2)	11 (16.7)	2.35 (1.14–4.83)	0.020 *	2.25 (0.99–5.08)	0.051	
Sigmoid colon	1 (6.3)	8 (12.1)	1.32 (0.60–2.94)	0.490	1.67 (0.74–3.78)	0.219	
Others	4 (25.0)	21 (31.8)	1.58 (0.88–2.82)	0.124	1.37 (0.76–2.48)	0.301	
Response at 6 months							1.57
Responders	16 (100.0)	42 (63.6)	ref				
Non-responders	0	24 (36.4)	1.96 (1.18–3.26)	0.009 *	1.78 (0.50–3.05)	0.696	
Progression, n (%)	0	36 (54.5)	2.05 (1.25–3.36)	0.004 *	2.79 (1.33–5.88)	0.007 *	1.77
Endocan, pg/mL	240.4 (180.4–383.7)	453.4 (336.9–559.9)	1.03 (1.01–1.04)	<0.001 *	1.03 (1.01–1.04)	<0.001 *	1.55
PDGF-DD, pg/mL	280.8 (229.7–396.1)	536.2 (381.6–647.1)	1.02 (1.01–1.03)	0.001 *	1.02 (1.01–1.04)	0.003 *	1.50
PDGF-CC, pg/mL	1439 (847–1614)	2872 (1752–3381)	1.02 (1.01–1.04)	0.005 *	1.02 (1.01–1.05)	0.018 *	1.58
					2 Log Likelihood = 459.6		

Data are mean ± standard deviation or median (IQR), or number (%). Age, gender, smoking, presence of comorbidity, chemotherapy type, metastasis area, mutation status, and anti-angiogenic agent type were adjusted in multivariable analyses. * *p* < 0.05 indicates statistical significance. For endocan, PDGF-DD, and PDGF-CC, HR (95% CI) are expressed per 10 pg/mL increase. Abbreviations: CEA, carcinoembryonic antigen; CI, confidence interval; HR: hazard ratio; PDGF-DD, platelet-derived growth factor-DD; PDGF-CC, platelet-derived growth factor-CC.

**Table 5:** Correlation analysis between serum endocan, PDGF isoforms, and tumor markers.

Variables	Endocan		PDGF-DD		PDGF-CC	
	r	*p*	r	*p*	r	*p*
PDGF-DD	0.417	<0.001 *	-	-	-	-
PDGF-CC	0.407	<0.001 *	0.551	<0.001 *	-	-
CA 19-9	0.319	0.048 *	0.230	0.285	0.250	0.255
CEA	0.302	0.006 *	0.247	0.287	0.212	0.315

* *p* < 0.05 indicates statistical significance. Abbreviations: CEA, carcinoembryonic antigen; PDGF-DD, platelet-derived growth factor-DD; PDGF-CC, platelet-derived growth factor-CC.

## Data Availability

The data that support the findings of this study are available on request from the corresponding author due to privacy and ethical reasons.

## Supplementary Materials

The following supporting information can be downloaded at https://www.mdpi.com/article/10.3390/ijms27062600/s1.
