# Supplementary material for: Prognostic Role of Endocan and Platelet-Derived Growth Factor Isoforms in Metastatic Colorectal Cancer
Source: Int J Mol Sci. 2026 Mar 12;27(6):2600. doi: 10.3390/ijms27062600 (PMC13026901; doi:10.3390/ijms27062600)
Supplement: Supplementary file 1 [file ijms-27-02600-s001.zip › ijms-4162777-supplementary Tables.pdf]

Supplement Table S1. Demographic and clinical characteristics of mCRC patients.

Variables	All mCRC patients<br>n = 82
Age, years	59.2 ± 11.6
≤65, n (%)	56 (68.3)
>655, n (%)	26 (31.7)
Female gender, n (%)	31 (37.8)
Smoking, n (%)	36 (43.9)
Comorbidities	30 (36.6)
CA 19-9, U/mL	26.0 (10.2 – 57.8)
CEA, ng/mL	7.0 (2.2 – 27.2)
Tumor location, n (%)	
Cecum	5 (6.1)
Right colon	6 (7.3)
Left colon	3 (3.7)
Rectum	36 (43.9)
Ascending colon	4 (4.9)
Rectosigmoid	12 (14.6)
Descending colon	1 (1.2)
Transverse colon	4 (4.9)
Hepatic flexure and rectum	2 (2.4)
Sigmoid colon	9 (11.0)
Chemotherapy, n (%)	
Oxaliplatin	64 (78.0)
Irinotecan	18 (22.0)
Metastasis area, n (%)	
Liver	53 (64.6)
Lung	30 (36.6)
Lymph node	43 (52.4)
Others	26 (31.7)
Mutations, n (%)	
KRAS	34 (41.5)
NRAS	13 (15.9)
BRAF	3 (3.7)
Bevacizumab, n (%)	57 (69.5)
Toxicity, n (%)	19 (23.2)
Response to CTx at 6 months	
Responders	58 (70.7)
Non-responders	24 (29.3)
Best response to CTx	
Responders	65 (79.3)
Non-responders	17 (20.7)
Outcomes, n (%)	
Progression	36 (43.9)
Mortality	66 (80.5)

Data are mean ± standard deviation or median (IQR), or number (%). Abbreviations: BRAF, B-Raf proto-oncogene serine/threonine kinase; CA 19-9, carbohydrate antigen 19-9; CEA, carcinoembryonic antigen; CTx, Chemotherapy; KRAS, Kirsten rat sarcoma viral oncogene homolog; mCRC, metastatic colorectal cancer; NRAS, neuroblastoma RAS viral oncogene homolog.

Supplement Table S2. Comparison of demographic and clinical characteristics according to best response to chemotherapy in mCRC patients.

Variables	Best response to CTx		p
	Responders n = 65	Non-responders n = 17	
Age, years	59.5 ± 11.4	58.3 ± 12.6	0.714
≤65, n (%)	45 (69.2)	11 (64.7)	0.721
>65, n (%)	20 (30.8)	6 (35.3)	
Female gender, n (%)	25 (38.5)	6 (35.3)	0.810
Smoking, n (%)	29 (44.6)	7 (41.2)	0.799
Comorbidities	24 (36.9)	6 (35.3)	0.901
CA 19-9, U/mL	25.0 (11.0 – 45.0)	35.0 (10.0 – 139.0)	0.496
CEA, ng/mL	7.0 (2.0 – 21.0)	15.0 (4.0 – 34.0)	0.045*
Tumor location, n (%)			
Cecum	5 (7.7)	0	0.712
Right colon	5 (7.7)	1 (5.9)	
Left colon	2 (3.1)	1 (5.9)	
Rectum	29 (44.6)	7 (41.2)	
Ascending colon	3 (4.6)	1 (5.9)	
Rectosigmoid	8 (12.3)	4 (23.5)	
Descending colon	1 (1.5)	0	
Transverse colon	2 (3.1)	2 (11.8)	
Hepatic flexure and rectum	2 (3.1)	0	
Sigmoid colon	8 (12.3)	1 (5.9)	
Chemotherapy, n (%)			
Oxaliplatin	51 (78.5)	13 (76.5)	0.702
Irinotecan	14 (21.5)	4 (23.5)	
Metastasis area, n (%)			
Liver	42 (64.6)	11 (64.7)	0.994
Lung	24 (36.9)	6 (35.3)	0.901
Lymph node	35 (53.8)	8 (47.1)	0.618
Others	20 (30.8)	6 (35.3)	0.721
Mutations, n (%)	31 (47.7)	6 (35.3)	0.360
KRAS	29 (44.6)	5 (29.4)	0.257
NRAS	12 (18.5)	1 (5.9)	0.206
BRAF	2 (3.1)	1 (5.9)	0.583
Bevacizumab, n (%)	48 (73.8)	9 (52.9)	0.096
Toxicity, n (%)	15 (23.1)	4 (23.5)	0.969
Response to CTx at 6 months			
Responders	58 (89.2)	0	<0.001*
Non-responders	7 (10.8)	17 (100.0)	
Outcomes, n (%)			
Progression	21 (32.3)	15 (88.2)	<0.001*
Mortality	49 (75.4)	17 (100.0)	<0.001*

Data are mean ± standard deviation or median (IQR), or number (%). *p<0.05 indicates statistical significance. Abbreviations: BRAF, B-Raf proto-oncogene serine/threonine kinase; CA 19-9, carbohydrate antigen 19-9; CEA, carcinoembryonic antigen; CTx, Chemotherapy; KRAS, Kirsten rat sarcoma viral oncogene homolog; mCRC, metastatic colorectal cancer; NRAS, neuroblastoma RAS viral oncogene homolog.

Supplement Table S3. Demographic and clinical parameters associated with disease progression.

Variables	Disease progression		Crude regression	
	No n = 46	Yes n = 36	HR (95% CI)	p
Age, years	58.8 ± 12.1	59.7 ± 11.0	1.00 (0.97 – 1.03)	0.853
≤65, n (%)	31 (67.4)	25 (69.4)	ref	
>65, n (%)	15 (32.6)	11 (30.6)	0.85 (0.42 – 1.75)	0.667
Female gender, n (%)	20 (43.5)	11 (30.6)	0.66 (0.32 – 1.34)	0.253
Smoking, n (%)	21 (45.7)	15 (41.7)	0.95 (0.49 – 1.86)	0.892
Comorbidities	16 (34.8)	14 (38.9)	1.10 (0.56 – 2.14)	0.790
CA 19-9, U/mL	26.0 (7.2 – 39.2)	26.0 (12.8 – 135.2)	1.01 (0.98 – 1.05)	0.946
CEA, ng/mL	7.0 (2.2 – 23.2)	18.0 (5.0 – 35.5)	1.03 (1.02– 1.07)	0.047*
Tumor location, n (%)				
Rectum	21 (45.7)	15 (41.7)	ref	
Rectosigmoid	6 (13.0)	6 (16.7)	1.29 (0.50 – 3.34)	0.600
Sigmoid colon	6 (13.0)	3 (8.3)	0.61 (0.18 – 2.09)	0.428
Others	13 (28.3)	12 (33.3)	1.39 (0.65 – 2.98)	0.392
Chemotherapy, n (%)				
Oxaliplatin	36 (78.3)	28 (77.8)	ref	
Irinotecan	10 (21.7)	8 (22.2)	1.12 (0.51 – 2.46)	0.780
Metastasis area, n (%)				
Liver	27 (58.7)	26 (72.2)	1.7 (0.82 – 3.54)	0.153
Lung	18 (39.1)	12 (33.3)	0.71 (0.35 – 1.44)	0.341
Lymph node	21 (45.7)	22 (61.1)	1.46 (0.75 – 2.86)	0.270
Others	12 (26.1)	14 (38.9)	1.48 (0.75 – 2.89)	0.255
Mutations, n (%)				
KRAS	22 (47.8)	12 (33.3)	0.62 (0.31 – 1.23)	0.172
NRAS	8 (17.4)	5 (13.9)	0.90 (0.35 – 2.32)	0.826
BRAF	1 (2.2)	2 (5.6)	1.54 (0.37 – 6.46)	0.556
Bevacizumab, n (%)	32 (69.6)	25 (69.4)	0.96 (0.47 – 1.96)	0.921
Toxicity, n (%)	10 (21.7)	9 (25.0)	1.07 (0.50 – 2.27)	0.865
Response to CTx at 6 months				
Responders	42 (91.3)	16 (44.4)	ref	
Non-responders	4 (8.7)	20 (55.6)	5.40 (2.73 – 10.69)	<0.001*
Best response to CTx, n (%)				
Responders	44 (95.7)	21 (58.3)	ref	
Non-responders	2 (4.3)	15 (41.7)	3.98 (2.03 – 7.81)	<0.001*
Mortality, n (%)	30 (65.2)	36 (100.0)	–	-

Data are mean ± standard deviation or median (IQR), or number (%). *p<0.05 indicates statistical significance. Abbreviations: BRAF, B-Raf proto-oncogene serine/threonine kinase; CA 19-9, carbohydrate antigen 19-9; CEA, carcinoembryonic antigen; CTx, Chemotherapy; CI, confidence intervals; HR, hazard ratio; KRAS, Kirsten rat sarcoma viral oncogene homolog; mCRC, metastatic colorectal cancer; NRAS, neuroblastoma RAS viral oncogene homolog.

Supplement Table S4. Demographic and clinical parameters associated with mortality.

Variables	Alive<br>n = 16	Deceased<br>n = 66	Crude regression<br>HR (95% CI)	 p
Age, years	58.5 ± 10.5	59.4 ± 11.9	1.01 (0.98 – 1.03)	0.542
≤65, n (%)	12 (75.0)	44 (66.7)	ref	
>65, n (%)	4 (25.0)	22 (33.3)	1.25 (0.75 – 2.10)	0.392
Female gender, n (%)	7 (43.8)	24 (36.4)	0.92 (0.55 – 1.52)	0.734
Smoking, n (%)	9 (56.2)	27 (40.9)	0.71 (0.44 – 1.17)	0.180
Comorbidities	8 (50.0)	22 (33.3)	1.04 (0.62 – 1.74)	0.895
CA 19-9, U/mL	25.5 (7.5 – 29.0)	26.0 (11.2 – 78.8)	1.01 (0.99 – 1.02)	0.377
CEA, ng/mL	3.5 (2.0 – 7.2)	9.5 (4.0 – 33.2)	1.03 (1.01 – 1.06)	0.007*
Tumor location, n (%)				
Rectum	10 (62.5)	26 (39.4)	ref	
Rectosigmoid	1 (6.2)	11 (16.7)	2.35 (1.14 – 4.83)	0.020*
Sigmoid colon	1 (6.3)	8 (12.1)	1.32 (0.60 – 2.94)	0.490
Others	4 (25.0)	21 (31.8)	1.58 (0.88 – 2.82)	0.124
Chemotherapy, n (%)				
Oxaliplatin	14 (87.5)	50 (75.8)	ref	
Irinotecan	2 (12.5)	16 (24.2)	1.5 (0.85 – 2.64)	0.163
Metastasis area, n (%)				
Liver	7 (43.8)	46 (69.7)	1.59 (0.94 – 2.70)	0.083
Lung	7 (43.8)	23 (34.8)	0.76 (0.46 – 1.27)	0.297
Lymph node	8 (50.0)	35 (53.0)	0.80 (0.49 – 1.31)	0.375
Others	6 (37.5)	20 (30.3)	0.80 (0.47 – 1.35)	0.400
Mutations, n (%)				
KRAS	6 (37.5)	28 (42.4)	1.05 (0.65 – 1.72)	0.833
NRAS	2 (12.5)	11 (16.7)	1.44 (0.74 – 2.80)	0.277
BRAF	1 (6.2)	2 (3.0)	1.03 (0.25 – 4.23)	0.965
Bevacizumab, n (%)	9 (56.2)	48 (72.7)	1.20 (0.69 – 2.07)	0.522
Toxicity, n (%)	3 (18.8)	16 (24.2)	1.03 (0.59 – 1.82)	0.908
Response at 6 months				
Responders	16 (100.0)	42 (63.6)	ref	
Non-responders	0	24 (36.4)	1.96 (1.18 – 3.26)	0.009*
Best of response, n (%)				
Responders	16 (100.0)	49 (74.2)	ref	
Non-responders	0	17 (25.8)	2.46 (1.38 – 4.39)	0.002*
Progression, n (%)	0	36 (54.5)	2.05 (1.25 – 3.36)	0.004*

Data are mean ± standard deviation or median (IQR), or number (%). *p<0.05 indicates statistical significance. Abbreviations: BRAF, B-Raf proto-oncogene serine/threonine kinase; CA 19-9, carbohydrate antigen 19-9; CEA, carcinoembryonic antigen; CTx, Chemotherapy; CI, confidence intervals; HR, hazard ratio; KRAS, Kirsten rat sarcoma viral oncogene homolog; mCRC, metastatic colorectal cancer; NRAS, neuroblastoma RAS viral oncogene homolog.
